# RETRACTION: Psychometric Validation of The Persian Version of the Ethical Awareness Scale for Nurses Working in Iranian Intensive Care Units

**DOI:** 10.1002/nop2.70559

**Published:** 2026-06-16

**RETRACTION**: S. Kolagari, R. Moradi, A. Milliken, H. Khoddam, “Psychometric validation of the Persian version of the ethical awareness scale for nurses working in Iranian intensive care units,” *Nursing Open* 11, no. 6 (2024): e2168, https://doi.org/10.1002/nop2.2168.

The above article, published online on 09 June 2024 in Wiley Online Library (wileyonlinelibrary.com), has been retracted by agreement between the journal Editor‐in‐Chief, Diana Baptiste; and John Wiley & Ltd. The retraction has been agreed due to concerns raised by the third party, which revealed inaccurate data in Table 1, Table 2, and Figure 1. The corresponding author collaborated in the investigation and confirmed the existence of errors in the published article on the basis of re‐evaluation by an independent statistical consultant. The editors consider the conclusions of this manuscript substantially compromised. The authors were informed of the retraction.

